# Etiology and clinical characteristics of pediatric acute fever among hospitalized children in an endemic malaria transmission area of Cameroon in Central Africa

**DOI:** 10.1371/journal.pone.0278407

**Published:** 2023-01-24

**Authors:** Calixte Ida Penda, Patricia Épée Eboumbou, Grace Ngondi, Jean Baptiste Hzounda Fokou, Christelle Véronique Pfoum, Ritha Mbono Betoko, Charlotte Eposse, Laurent-Mireille Endale, Francine Same Bebey, Carole Else Eboumbou Moukoko

**Affiliations:** 1 Department of Clinical Sciences, Faculty of Medicine and Pharmaceutical Sciences, University of Douala, Douala, Cameroon; 2 Pédiatric Unit, Douala Laquintinie Hospital, Douala, Cameroon; 3 Pédiatric Unit, Douala General Hospital, Douala, Cameroon; 4 Pédiatric Unit, Bonassama District Hospital, Douala, Cameroon; 5 Department of Biological Sciences, Faculty of Medicine and Pharmaceutical Sciences, University of Douala, Douala, Cameroon; 6 Biology Lab, Douala Laquintinie Hospital, Douala, Cameroon; 7 Department of Pharmaceutical Sciences, Faculty of Medicine and Pharmaceutical Sciences, University of Douala, Douala, Cameroon; 8 Center for Respiratory Diseases, Douala Laquintinie Hospital, Douala, Cameroon; 9 Internal Medicine Department, Douala Laquintinie Hospital, Douala, Cameroon; 10 Malaria Research Unit, Centre Pasteur Cameroon, Yaoundé, Cameroon; 11 Laboratory of Parasitology, Mycology and Virology, Postgraduate Training Unit for Health Sciences, Postgraduate School for Pure and Applied Sciences, University of Douala, Douala, Cameroon; George Washington University School of Medicine and Health Sciences, UNITED STATES

## Abstract

Acute fever in the majority of children in resource-limited countries is attributable to malaria and often treated without laboratory evidence. The aim of the study was to characterize acute pediatric infectious fevers (APIF) in the pediatric department of the Douala Laquintinie Hospital. A cross-sectional study was conducted among children aged 2 months to 15 years who were admitted with an acute fever (anal temperature ≥ 37.5°C less than 5 days in infants and 7 days in adolescents). 200 children were included and followed up during their hospitalization. The mean age was 3.7 (IQ25-75: 1–4.6) years. More than 3 out of 5 patients (62.5%) came from another health facility and anemia accounted for 29% of the reasons for consultation associated with fever. The main symptoms were vomiting (28%), cough (26%), convulsions (21%) and diarrhea (20%). Skin-mucosal pallor (43.0%) and hepatosplenomegaly (26.0%) were the most common physical signs encountered. Among febrile children, 116/200 (58%) were infected with at least 1 pathogen, and 1/200 (0.5%) had a fever of unknown etiology. Malaria (53% vs 80.5% presumptive) associated with anemia (95.3% of cases) was the most common pathology associated with APIF, followed by pneumonia (19.5%), meningitis (11.5%) and urinary tract infections (10% vs 54.5% presumptive). Malaria was over-diagnosed on admission and over-treated as well as urinary tract infection. A better understanding of common pathogens carriage, a better capacity for improved diagnosis and a better applied clinical algorithm for febrile illnesses in children are needed.

## Introduction

Fever is a nonspecific defense reaction of the body in response to the action of various triggers including pathogens and some non-living factors such as hormones and drugs. It is the warning signal for a rise of temperature above 38°C and the most common clinical sign in children under 5 in Africa [1]. Fever from an infectious origin is the most frequent and can cause complications such as febrile convulsions or even lead to death [2–6]. Acute fever is defined as lasting less than five days in infants less than 2 years old while in elder children fever remains acute for up to three weeks [7, 8]. The incidence of pediatric acute fevers (PAF) varies among studies. The global burden of febrile illness and the contribution of many pathogens inducing fever are difficult to quantify and characterize. In sub-Saharan Africa, fever is a common symptom [2] and febrile disease is a major cause of illness and death [3]. A study conducted in 42 countries in sub-Saharan Africa report an incidence of 655.6 million fever episodes in children under five years old in 2007 [4] and in Cameroon, in the best of our knowledge, no data is reported in the literature. Acute unexplained fevers (AUF) represent 14% of PAF, they can hide a severe bacterial infection (occult bacteremia, bacterial meningitis, acute pyelonephritis.) which in the absence of early and adequate treatment, can lead to serious complications [9, 10].

Despite the similar clinical features of a wide spectrum of potential etiologies of PAF [11, 12], studies highlight that the most common cause of PAF in our setting remains malaria. Indeed, despite the reduction in malaria-related mortality (30% in 2012 to 12.4% in 2016) among children under 5 years old [5–8] in Cameroon, malaria remains significant. Data from the literature nevertheless reports an overestimation of the number of cases of malaria and studies reported that more than half of febrile children are considered not to have malaria [11–16]. In addition to the prevalence of antimicrobial resistance among bacterial isolates which continues to increase [17, 18], the World Health Organization (WHO) recommends confirming *Plasmodium* infection in febrile children by a laboratory examination before initiating the treatment and systematically making the differential diagnosis of fever [19, 20].

Diagnosing acute pediatric infectious fever (APIF), specifying the cause and initiating symptomatic treatment followed by specific etiological treatment are the main steps in the fever management algorithm. There is no diagnostic standard for APIF, and it is difficult to discern the different possible etiologies of a febrile illness with the medical history and physical examination without references, clinical studies and biological confirmation. The diagnostic approach in APIF should include in addition to the history of the febrile symptom and repeated physical examinations, additional paraclinical examinations such as procalcitonin (PCT), C-reactive Protein (CRP), urine dipstick and the analysis of the cerebrospinal fluid (CSF) or even an X-ray which offers the possibility of identifying different etiology of APIF [21–29].

Studies on the multiple potential causes of fever are rare and the pathogen remains unidentified for many patients [30–32] due to the inadequacy or absence of laboratory equipment and reagents in health facilities in the resource-limited countries and where several co-infecting organisms exist [33]. To the best of our reading, no data on the etiologies and clinical characteristics of APIF in Cameroon is reported in the literature. The only one available was carried out since 1996 in Yaoundé [34]. The present work aimed to establish an etiological profile and clinical characteristics of APIF in the pediatric department of the Douala Laquintinie Hospital (DLH).

## Materials and methods

### i) Study design and study population

A prospective cross-sectional study was carried out from March 1^st^ to May 31^st^, 2019, in 2 units (general pediatric and emergency-intensive care) of the DLH pediatric department. The DLH is a second category care and teaching hospital located in the city of Douala with specialist as pediatricians, biologists. The pediatric unit in DHL is managed by 3 pediatricians, 5 general practitioners and nurses;

With an estimated average annual population growth rate of 5% over the past 30 years and a current population of around 2,500,000 inhabitants, Douala is, therefore, the city with the largest population in Cameroon. The annual population growth rate is estimated to an average of 5% over the past 30 years.

This study was conducted following ethics directives related to research on humans in Cameroon. The study received ethical clearance from the Institutional Committee of Ethics for Research for Human Health of the University of Douala (N° 1763 CEI-UDO / 06/2019 / T) and, an administrative agreement (N° 1134 / AR / MINSANTE / DHL / CM) was obtained from the DLH. Before enrollment and the administration of the questionnaire, subjects were informed of on the purpose and process of the investigation (background, goals, methodology, study constraints, data confidentiality, and rights to opt-out from the study), and a signed informed consent was obtained from the children’s parents/guardians in accordance with the Helsinki Declaration. All patients were free treated in accordance to the treatment guidelines from the Cameroon National Malaria Control Program.

Assuming an estimated fever prevalence of 15% in 2014 in Cameroon, the minimum sample size of 196 children would be needed using Cochran’s formula [35]. Convenience and non-probabilistic sampling are applicable in the study when members of the population are convenient to sample. To reduce selection and information biases, patients were enrolled consecutively, voluntary, anonymously and without remuneration.

The target population was all children aged 2 months to 15 years who attended DLH in consultation. Eligibility for inclusion was defined for any children attending pediatric emergency department with an anal temperature ≥ 37.5°C and/or presenting an infectious syndrome, i.e. at least a Systemic Response Inflammatory Syndrome (SIRS). SIRS was defined as a combination of at least 2 signs (including body temperature > 38°C or <36°C and/or a heart rate > 90 beats/min and/or a respiratory rate > 20/min and/or a hyperventilation resulting in PaCO_2_ < 32mmHg (<4.3kPa) in ambient air) [36]. Fever (temperature ≥ 38°C in the anal record) was said to be acute when it had been present for less than five days in infants (≤ 24 months) and less than seven days in children (between 24 months and 15 years) [37]. Anemia was determined according to age (haemoglobin level < 11 g/dL in children under 5 years old, < 11.5 g/dL in children aged 5 to 11 years 11 months and < 12g/dL in children aged 12 to 14 years 11 months [38].

The administered questionnaire was done following a one-week pre-test with 15 parents/guardians in another hospital structure to assess: i) the understanding and acceptability of the parents/guardians in the study and ii) for standardizing and homogenizing data collection in the two units. The pre-tested structured questionnaire was administered to parents/guardians during a 15-minute one-to-one interview to collect data on the reason for consultation, disease history and patient history. After the pre-test, some changes were made. The interview questions have been worded in such a way that they do not influence the participants in their answers. The questionnaire was administered independently on the same day by two investigators interviewing parents/guardians for 10 minutes to estimate the inter-reproducibility of the interviewer.

### ii) Study questionnaire

These were open (OEIQ) and closed (CEIQ) interview questions, including single answer and, multiple-choice questions. Data collection sheets were used to collect data on i) socio-demographic characteristics, ii) anthropometric parameters (restricted to weight, heart and respiratory rate), iii) patient history (including the previous episode of malaria within 15 days of preceding the consultation, previous pathology, vaccination, comorbidities, the notion of recent blood transfusion), iv) clinical symptoms suggesting an infectious state (asthenia, diarrhea, urination burns, prostration, hematuria, convulsion, cough, abdominal pain, vomiting), v) the physical signs of the different systems (disturbance of consciousness, respiratory distress, jaundice, hepatomegaly, splenomegaly, anemia, bladder), vi) paraclinical data (biological assessment: complete blood count-CBC, rapid diagnostic test-RDT and thick blood smear (TBS) for the diagnosis of malaria, urine dipstick, blood assay of Pro-Calcitonin-PCT and Creative protein-CRP). When the urine dipstick was positive, the physician ordered a cytobacteriological examination of the urine.

Patient medical records were also reviewed for results relating i) to patient history, ii) performing the blood culture, iii) cytobacteriological examination of urine (CBEU), iv) cerebrospinal fluid analysis, v) chest x-ray previously requested by the prescriber and made according to the clinical context (at the expense of the parents) and, vi) therapeutic data.

### iii) Paraclinical examinations

The initial diagnostic test used was the RDT (Selex On Mal/Pf/Pan test strip and SD BIOLINE Malaria Ag.Pf) for qualitative detection from whole blood. Microscopy was performed to estimate the parasite density on the different samples and subsequently, 10% of the slides were checked by an experienced technician. The *Plasmodium* parasites densities (PD) expressed as the number of parasites per microliter (μL) of blood was determined based on the number of parasites per 500 leukocytes on a TBS, assuming total leukocyte counts of 8,000 cells/microL of whole blood. Severe malaria is characterized by the presence of *P*. *falciparum* infection, clinical manifestations of malaria (fever with or without hyperthermia at the time of consultation, asthenia, headache, nausea or vomiting) and the presence of either or more of the signs defining severe forms of malaria defined by WHO [37].

PCT (cut-off value> 0.05) is produced specifically in the event of microbial infection indicated when bacterial, parasitic or fungal infections are suspected and its value compared to that of CRP (cut-off value ≥ 6 mg/L) is its rapid rise during bacterial infection [39]. The urine dipstick (CombiScreen) has been used to detect and monitor urinary tract infections, diabetes, and renal glucose levels [40].

### iv) Statistical analysis

Categorical variables were expressed as frequencies, while numeric variables were presented as means +/- standard deviation (SD) or 95% CI (95% confidence interval) if they were normally distributed. Fisher’s exact test was used to compare qualitative variables. After checking the non-Gaussian distribution, the nonparametric Mann-Whitney U test was used to compare the variables between two independent groups. Only p values <0.05 were considered significant. All statistical analyses were performed using GraphPad Prism5 software (San Diego, CA, USA) and Statistical Package for Social Sciences (SPSS) version 20 software.

## Results

### Epidemiological characteristics

**Fig 1:**
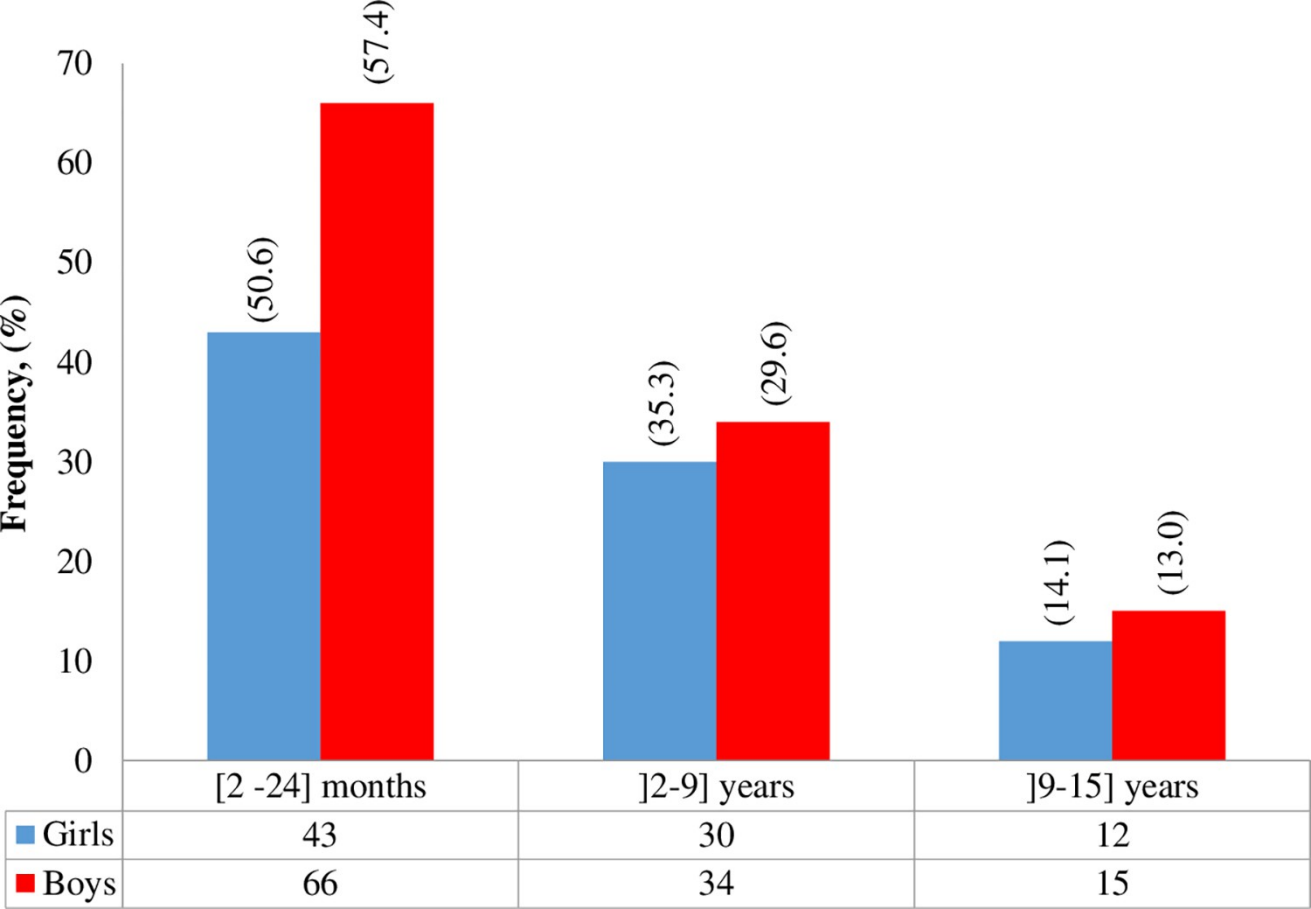
Description of the population studied according to gender and age groups.

A total of 273 patients were admitted for suspected fever, of which 200 met the inclusion criteria with an APIF, for an inclusion rate of 73.3%. Most of the children were boys (115; 57.5%) with a sex ratio of 1.38 (Fig 1).

More than half (54.5%) of patients were infants (2months-24months) and 27 (13.5%) were adolescents (10 years—15 years). The mean age was 3.7 (IQ25-75: 1–4.6) years and did not differ statistically between girls and boys (p = 0.481).

More than the majority (112; 56.0%) of parents/guardians was employed, of which 21.0% had fixed incomes. Most mothers / guardians (52.9% vs 37.4% for men / guardians) were unemployed (OR = 1.88; p = 0.003; 95% CI: 1.10–3, 33). More than half (58.0%) of parents had secondary education and 34 (17.0%) primary education.

### Clinical history of the study population at admission

The consultation time after the onset of symptoms was 3 days for most children (86; 43.0%) and 7 days for 32.5% of cases. Three over 5 children (62.5%) came from another health facility, no significant difference was observed by age group or gender. Out of 200 patients included, 161 (80.5%) slept under a long-lasting impregnated mosquito nets (LLINs) and 43 (76.7%) had full vaccination coverage from the Expanded Immunization Program.

**Fig 2:**
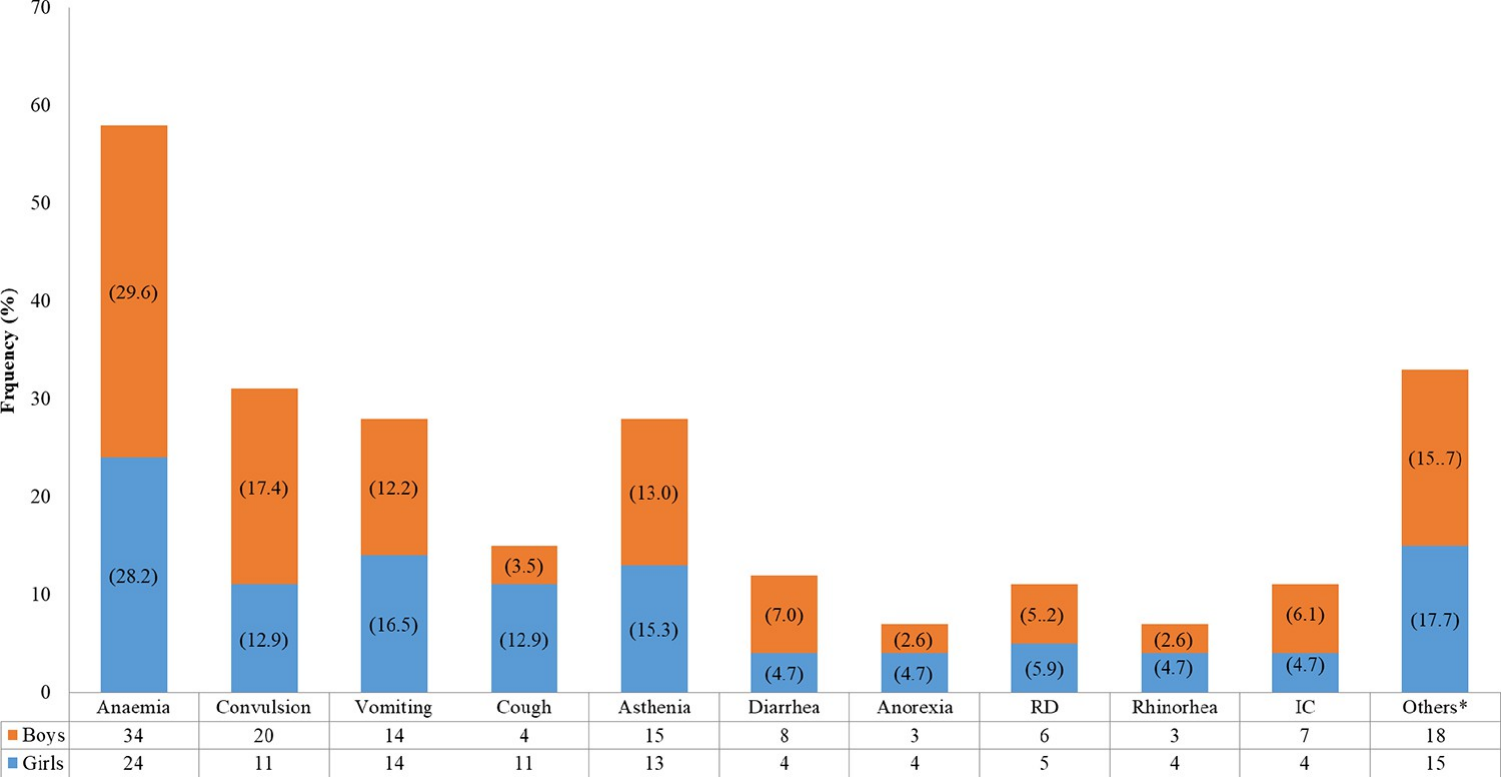
Frequency of reasons for consultation according to gender. Note. RD, Respiratory Distress; IC, impaired consciousness; Others*, headache + prostration + weight loss + abdominal pain + joint pain + rashes + ingestion of caustics.

The main reasons for patients consultation were fever associated with anemia (29.0%) followed by convulsions (15.5%), vomiting (14.0%) and asthenia (14.0%) (Fig 2).

### Physical examination: Vital parameters and clinical features

The patient’s temperature was ranging from 35 to 41°C with a mean of 39.1°C (95% CI: 39.0–39.3). 34.5% (69) had a temperature ≥ 39.5° C (Table 1).

**Table 1:** Clinical characteristics of the study population.

Variables	Boys	Girls	Total	p
	N = 115	N = 85	N = 200	
**Vital parameters**				
***Mean temperature (Sd)*. *°C***	39.1 (0.69)	39.1 (0.94)	39.1 (0.81)	0.170
[35–38]	1 (0.87)	2 (2.35)	3 (1.50)	
[38–39]	65 (56.52)	44 (51.76)	109 (54.50)	
[39–40]	42 (36.52)	31 (36.47)	73 (36.50)	
[40–41]	7 (6.09)	8 (9.41)	15 (7.50)	
***Respiratory Frequency***				0.624
Normal	51 (44.4)	33 (38.8)	84 (42.0)	
Tachypnea	53 (46.1)	45 (52.9)	98 (49.0)	
Bradypnea	11 (9.6)	7 (8.2)	18 (9.0)	
***Cardiac frequency***				0.379
Normal	69 (60.0)	46 (54.1)	115 (57.5)	
Tachycardia	46 (40.0)	38 (44.7)	84 (42.0)	
Bradycardia	0 (0.0)	1 (1.18)	1 (0.50)	
**General presentation**				
Asthenia	107 (93.0)	74 (87.1)	181 (90.5)	0.119
Anorexia	63 (57.8)	47 (55.3)	110 (55.0)	0.529
Weight loss	9 (7.8)	14 (16.5)	23 (11.5)	0.048*
**Functional signs**				
Digestive	52 (45.2)	33 (38.8)	85 (42.5)	0.224
Neurologic	48 (41.7)	33 (38.8)	81 (40.5)	0.394
Respiratory	29 (25.2)	31 (36.5)	60 (30.0)	0.060
Ear-Nose-throat	18 15.7)	15 (17.7)	33 (16.5)	0.425
Urinary	8 (7.0)	8 (9.4)	16 (8.0)	0.579
**Physical signs**				
Icterus	12 (10.43)	8 (9.41)	20 (10.0)	0.504
Cutaneo-muquous palor	49 (42.6)	37 (43.5)	86 (43.0)	0.906
Hepatomegaly	16 (13.9)	7 (8.24)	23 (11.5)	0.154
Splenomegaly	20 (17.4)	9 (10.6)	29 (14.5)	0.125
Respiratory Distress	9 (7.8)	9 (10.1)	18 (9.0)	0.333

Data are number and/or proportion (%), unless otherwise indicated; *, Sd: Standard deviation; *, p-value showing the statistical significant.

Normal respiratory rate was found in 42.0% of case and 9.0% had bradypnea (respiratory rate below the normal value) determined by age [41] and 57.5% of the patients had a normal heart rate. Tachycardia (heart rate above normal value) was found in 42.0% of patients while 1 patient presented with bradycardia (heart rate above normal value) determined by age [41].

Many patients presented with asthenia (90.5%) and/or anorexia (55%). Among the patients presenting functional signs, 156 (78.0%) had at least one functional sign (min-max: 1–4), digestive and neurological signs were found in 85 (42.5) and 81 (40, 5%) patients respectively. Among the digestive signs, vomiting (65.9%) and diarrhea (47.1%) were the most frequent signs. Seizures (21%) and prostration (15.5%) were the most common neurologic signs and 3.5% of children presented with impaired consciousness. Hematuria was found in 68.7% of the children. Cough (86.7%) and dyspnea (28.3%) were the most common respiratory signs. Among the physical signs associated with fever, mucocutaneous pallor was present in the majority of patients (43.0%). Epigastric pain, dehydration and abdominal pain was also found in the patients.

### Presumptive diagnosis on admission and treatment received on admission

On admission, malaria was suspected in 80.5% of the children, of which 93.8% would have severe malaria defined as malaria-associated mainly with anemia (Table 2).

**Table 2:** Presumptive diagnostic and treatment at initiation.

Presumptive diagnostic	Gender				Treatments		
	**Boys**	**Girls**	**Total**	**p**	**Antipyretics**	**Antibiotics**	**Antimalaria**
	**N = 115**	**N = 85**	**N = 200**				
**Malaria**	89 (77.4)	72 (84.7)	161 (80.5)	0.207	161 (100)	99 (61.5)	143 (88.8)
simple	7 (7.9)	3 (4.2)	10 (6.2)				
				0.265			
severe	82 (91.1)	69 (91.8)	151 (93.8)				
**Pneumonia**	23 (20.0)	22 (25.9)	45 (22.5)	0.208	45 (100.0)	38 (84.4)	8/10 (80.0)*
**Gastroenteritis**	12 (10.4)	6 (7.1)	18 (9.0)	0.220	19 (100)	8 (42.1)	7/11 (63.6)*
**Meningitis**	22 (19.1)	15 (17.6)	37 (18.5)	0.469	37 (100)	34 (91.9)	1/1 (100.0)*
**Urinary infection**	65 (56.5)	44 (51.7)	109 (54.5)	0.300	109 (100)	65 (59.6)	2/3 (66.7)*
**Acute otitis media**	3 (2.6)	2 (2.35)	5 (2.5)	0.640	5 (100)	4 (80.0)	0 (0.0)*
**Others** ^**$**^	36 (31.3)	27 (31.7)	63 (31.5)	0.462	/	/	/

Data are number and/or proportion (%); ^$^, Different systems impaired (cardiac, digestive, hematological, neurological, Ear-Nose-throat, cutaneous) + sepsis + poisoning; *, Results obtained on the group of patients not presenting a suspicion of malaria in the group of the studied variable.

Urinary tract infection and pneumonia were suspected in 54.5% and 22.5% of patients respectively. All children received an antipyretic and the majority an antimalarial (88.8%). From patients with suspected pneumonia or gastroenteritis in the absence of suspected malaria, 80% and 63.6% respectively received an antimalarial. Of 99 children admitted with suspected *Plasmodium* infection who received antibiotics on admission, 15.1% had no other suspicion of infections requiring antibiotic therapy. Meningitis was suspected in 37 (18.5%) of whom 91.9% received antibiotics on admission. All 8 children admitted with suspected gastroenteritis and who received antibiotics were aged from 2 to 24 months.

### Laboratory assessment, etiological diagnosis and clinical course

From 200 patients, 38.5% had a positive RDT confirmed by a positive HD and 31.5% of the children had no biologically confirmation Plasmodium infection (Table 3).

**Table 3:** Laboratory examinations of patients at the initiation.

Tests performed	N (%)	
**RDT/TBS (**N = 200)	RDT^+^/TSB^+^	77 (38,5)
	RDT^+^/TSB^-^	29 (14,5)
	RDT^-^/TSB^+^	31 (15,5)
	RDT^-^/TSB^-^	63 (31,5)
**Haemoglobin level** (N = 200)	Severe anemia	73 (36,5)
	Moderate anemia	78 (39,0)
	Low anemia	24 (12,0)
	No anemia	25 (12,5)
**UD** (N = 200)	UD^+^	27 (13,5)
**UD/CBEU** (N = 16)	UD^+^ /CBEU^+^	5 (31,3)
	UD^+^/CBEU^-^	11 (68,8)
**CRP/PCT** (N = 200)	CRP^+^/PCT^+^	96 (48,0)
	CRP^-^/PCT^+^	20 (10,0)
	CRP^+^/PCT ^-^	50 (25,0)
	CRP^-^/PCT^-^	34 (17,0)
**CFA cytology** (N = 31)	CFAc^+^ (≥ 10 leucocytes /mm3)	18 (58,1)
	CFAc- (≤10 leucocytes /mm3)	13 (41,9)
**CFA bacteriology** (N = 31)	CFAb^+^	2 (6,4)

Data are number and/or proportion (%); RDT, rapid diagnostic test; TBS, thick blood smear; UD, urine dipstick; CBEU, cytobacteriological examination of urine; CRP, C-reactive protein; PCT, Procalcitonine; CFA, cerebrospinal fluid analysis

In this study, 87.5% of patients admitted with fever had anemia, of which 36.5% had a severe form. The urine dipstick was positive in 13.5% of patients and 59.3% were able to perform a CBEU among which 31.3% were positive and the main germ found was *Escherichia Coli*. CFA for bacterial infection was positive in 58% of the patients. A mismatch between CRP and PCT as a marker of infection was observed in 70 patients, of which 10% with CRP^-^/PCT^+^ and 25% with CRP ^+^/PCT^-^. The CFA cytology was in favor of bacterial meningitis in 58% of children out of 31 samples taken and the *Streptococcus pneumoniae* germ was found in 6.5% of patients.

**Fig 3:**
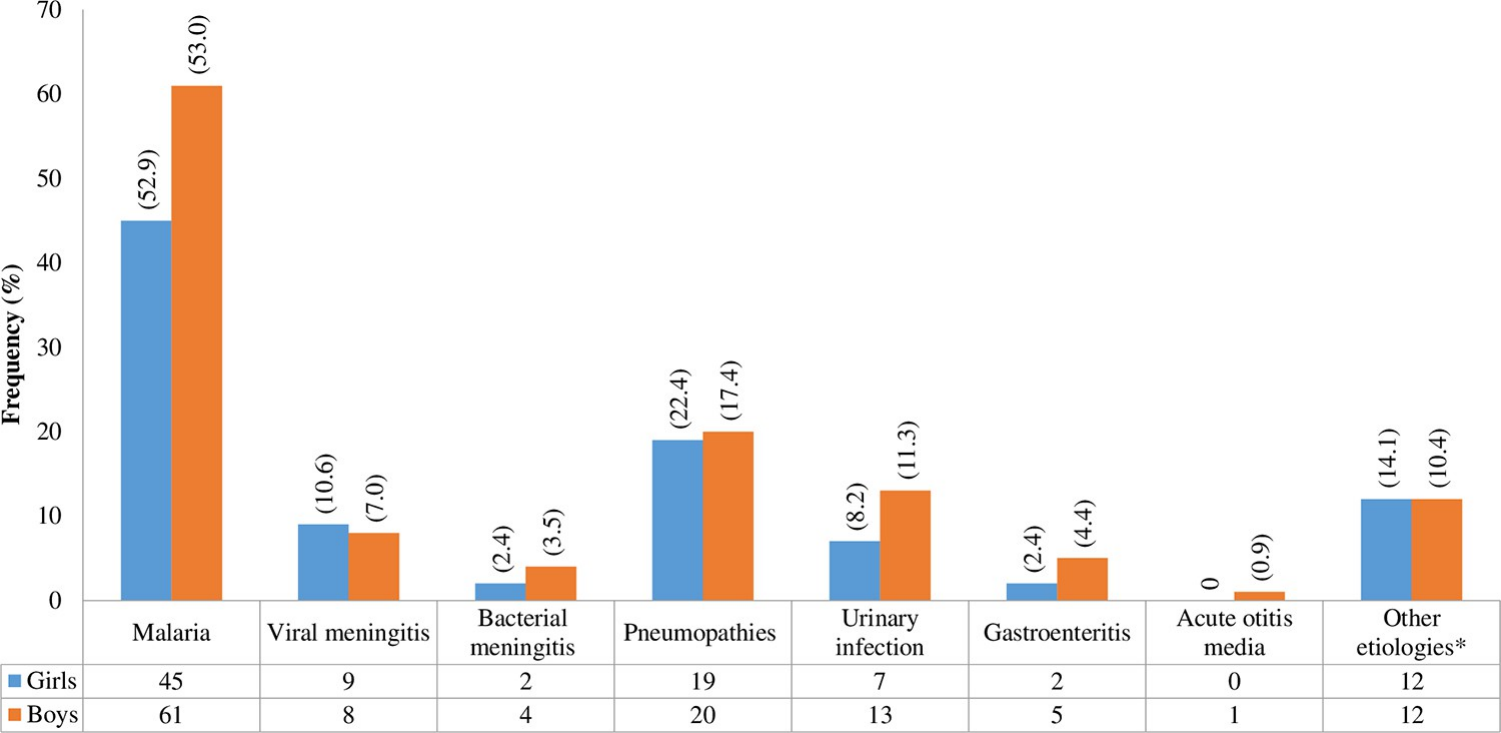
Confirmed diagnostic. Note. *, abscess; viral conjunctivitis; fever with unknown origin; bypass drain infection; osteoarticular infection; moderate/severe/acute malnutrition; myositis sepsis; esophageal stenosis; pulmonary tuberculosis associated with urinary infection.

Based on the laboratory tests, the treatment administered and the favorable clinical outcome of the patients, the diagnosis retained was malaria in 53% of the patients, of whom 95.3% had malaria associated with anemia (Fig 3).

Among patients, 22.7% had malaria associated with another infection (intestinal obstruction, erythematous angina, left blepharitis, urinary tract infection, meningitis, skin drug eruption, probable liver disease, pancytopenia due to G6PD deficiency, pneumonia, rhino-bronchitis, sepsis with a cutaneous call point, moderate acute malnutrition, and renal failure). Lung disease was present in 19.5% of patients with 1 case associated with severe acute malnutrition, urinary tract infections in 10% of patients and meningitis in 11.5% of patients.

Almost all patients cured, 6 (2%) children died and among them 2 children died few hours after their arrival in the hospital and 2 were still in hospitalization for complications not resolved during the study period.

## Discussion

The objective of this work was to study the etiologies of infectious fevers to identify the main etiologies of these fevers in children in Cameroon. To our knowledge, this study is the first published to report on febrile infections other than that linked to malaria in children in Cameroon. This study is also one of the few studies carried out in health facilities in Africa to date [24, 30, 42, 43].

In three months, 200 children admitted with fever in DLH were followed. The mean age of the patients was 24 months (12–180 months) and 57.5% were male and infants. This population distribution is comparable to that reported in other previous studies conducted on the etiology of fevers in Ivory Coast, Cameroon, Burkina Faso, Senegal, and Tanzania [24, 30, 42, 43]. The predominance of the male gender could be explained by a greater susceptibility of the said gender at extreme ages of life to morbid phenomena [44].

According to studies in Burkina Faso, Kenya and Cameroon, the average time taken by parents to bring their children for consultation after the onset of fever symptoms is 2 to 3 days [42, 44, 45]. Our study is consistent with previous studies, where the average delay was 4 (2–7) days and 42.5% of the children arrived for a consultation on average 2–3 days after the onset of symptoms and 32, 5% arrived after 7 days. Limited financial resources, self-medication, distance from the health structure and especially the long therapeutic course of sick children are obstacles to rapid recourse to hospital care [46]. In our population, 62.5% came from another health facility regardless of the group of children (infant, child, adolescent) while Doumbia et al., in 2001 [46] and Ouonogo et al., in 2003 [47] reported that 90% of the consulted children at the CHU-Gabriel Touré in Mali came directly from their homes. Given the large number of health centers in the city of Douala which are easily accessible to the population and at a lower cost, it is understandable that the time of arrival to the HLD although it is a referral hospital is late. Most children (94%) lived with progressive symptoms before hospitalization and 2 children died within hours upon arrival to Hospital.

As reported in previous studies, the main reason for consultation encountered was fever, it is mainly due to the high frequency of febrile illnesses in children and their frailties. It was mainly associated with anemia (34%), seizures (15.5%), asthenia (14%) and vomiting (14%). Previous studies conducted in Burkina Faso in 2009 and Cameroon in 2010 also report a high prevalence of anemia (74%) in febrile children [48, 49]. On the other hand, the cough (48%), vomiting (15.9%) and diarrhea (10.5%) trio were reported in other studies conducted in Kenya in 2015, Senegal in 2016 and Burkina Faso in 2018 [42, 44, 50]. The high prevalence of anemia as the main reason for consultation in our population reflects the high rate of anemia in the infantile population of Cameroon estimated at 57.6% [51]. Nevertheless, 33.5% of our patients had a previous pathology that could explain the fever and 23.2% of patients under 1-year-old had an incorrect vaccination status. This highlights the particular importance that must be given to the patient history analysis during diagnosis establishment when facing a fever.

The majority of the children had presented a digestive sign (42.5%) and/or a neurological sign (40.5%) on admission with vomiting (27%), cough (26%) and convulsions (21%) as main signs. This is in accordance with the Cameroon Demographic and Health Survey conducted in 2011 which reports that diarrhea and respiratory infections were the most important health problems in children [52]. Similar results from Kiemde et al., in Senegal reported a significant prevalence of cough (48.9%) followed by diarrhea (38.8%) [50] and vomiting (17.9%) in Burkina Faso [42].

The major physical signs associated with the fever were mucocutaneous pallor (43%) and in particular conjunctival pallor (40.5%) and that of the skin appendages (41.5%), hepatosplenomegaly (26%), signs of respiratory distress (9%) and crackles on auscultation (8.5%). These signs were as well as reported previously in Kenya [44] that report mucocutaneous pallor in 28.4% of cases, respiratory distress (11.4%), hepatosplenomegaly (2%) and crackles on auscultation (2%) in febrile children.

Based on laboratory and paraclinical examinations, 77.7% of fevers were attributable to an infectious pathology as reported in other countries in Africa [49, 53]. 53.0% of the children had confirmed malaria biologically associated or not with another pathology and 95.3% of them had severe malaria. Plasmodium infection has also been reported as the leading cause of fever in other studies although the frequencies are lower than that reported here (between 27.4% and 49.71%) [24, 42, 44]. While study from Tanzania in 2014 [31] reported pneumonia as the primary etiology of APIF in pediatrics patients, it was rather the second cause of fevers in the present study and this goes in the same line with other studies [44, 50]. The high frequency of pneumonia as the primary cause of APIF ahead of malaria in Tanzania can be explained by the drastic decline in malaria transmission observed since 2000 compared to other countries across sub-Saharan African countries and this might be the result of the large-scale implementation of effective control measures following a drastic increase in funding [14, 54–56].

Although severe malaria is the most common cause of APIF in hospitalized children in this study, it was over-diagnosed on admission (80.5% presumptively versus 53% etiologic diagnosis) and over-treated with antimalarials as well as with antibiotics. More than 1/3 of patients had received antimalarial treatments when they presented a negative TBS and a negative RDT and 15.1% of children with suspected *Plasmodium* infection and under antibiotics upon admission had no other suspected infections requiring antibiotic therapy. The fact that 3 out of 5 patients came from another health facility where they had probably received antimalarials could explain the negativity of TBS and the low density of the parasitemia at the time of sampling as in previous study [45]. In the same study, of the 267 patients who used a therapeutic recourse before admission, 62.6% had just one therapeutic itinerary and 37.8% had two therapeutic itineraries [45]. Prescribing antimalarial drugs for patients without parasitemia is well known, and many studies revealed similar practices [57, 58]. Usually, prescribers tend to err on the side of caution to offer treatment malaria endemic countries even when malaria tests are negative while several studies have shown that withholding treatment in cases where malaria tests are negative is safe [59, 60]. 21% of patients had received antibiotic therapy while they also presented a negative MDT reflecting the behavior of practitioners whose tendency is to initiate probabilistic antibiotic therapy in the face of a probable infection while awaiting examination results, even in young patients. A study conducted in pharmacies in Douala in the same study area, showed that 39.3% of antibiotics purchased by parents were intended for children under 15 years old and, around 60% of antibiotics dispensed were for children aged less than 5 years [61]. In addition, gastroenteritis was found in 19 patients, of whom 17 were infants. The viral etiology of enteritis in this age group has been confirmed by a meta-analysis rendering the use of antibiotics unnecessary [62]. In previous study conducted in the same hospital the most frequently used drugs were analgesics/antipyretics, antimalarial drugs and antibiotics [45]. In patients with no malaria parasites, the treating physicians seeing no characteristic clinical symptoms rather appears to rigorously follow the WHO guidelines for treating malaria in endemic malaria whilst also exploring treatment for other pathologies which may be co-existing. In fact, the WHO guidelines highlights the similarities and co-existence of malaria, pneumonia and septicaemia and recommends co-prescription of analgesics, antibiotics and haematinics even before laboratory results are obtained in children. This may also explain the relatively high concomitant prescription of antibiotics and others drugs in this study among patients with febrile illness and in another studies [45, 63, 64]. Moreover, analgesic and hematinic drugs are routinely prescribed for children to treat fever and anemia, respectively.

## Conclusion

This study highlights that malaria, pneumonia, and urinary tract infections were the main etiologies of fever in these patients in our setting. However, there was great variability between presumptive and definitive diagnosis, as the examinations for the necessary diagnosis of fever should primarily be guided by clinical history and relevant laboratory tests to improve fever management and reduce its frequency, incidence and health costs.

## Abbreviations

APIF: acute pediatric infectious fevers
AUF: acute unexplained fevers
CBC: complete blood coun
CBEU: cytobacteriological examination of urine
CEIQ: closed interview questions
CI: confidence interva
CRP: C-reactive protein
CSF: cerebrospinal fluid
CFA: cerebrospinal fluid analysis
DLH: Douala Laquintinie Hospita
LLINs: long-lasting impregnated mosquito nets
OEIQ: open interview questions
PAF: pediatric acute fevers
PCT: procalcitonin
PD: *Plasmodium* parasites densities
RDT: rapid diagnostic test
SD: standard deviation
SIRS: Systemic Response Inflammatory Syndrome
TBS: thick blood smear
UD: urine dipstick
WHO: World Health Organization
